# Diffuse intrinsic pontine glioma cells are vulnerable to low intensity electric fields delivered by intratumoral modulation therapy

**DOI:** 10.1007/s11060-019-03145-8

**Published:** 2019-03-09

**Authors:** Andrew Deweyert, Erin Iredale, Hu Xu, Eugene Wong, Susanne Schmid, Matthew O. Hebb

**Affiliations:** 1 Department of Anatomy and Cell Biology, Schulich School of Medicine and Dentistry, Western University, London, ON Canada; 2 Department of Medical Biophysics, Schulich School of Medicine and Dentistry, Western University, London, ON Canada; 3 Department of Clinical Neurological Sciences, Schulich School of Medicine and Dentistry, Western University, London, ON Canada

**Keywords:** Electrotherapy, Glioma, Pediatric, Brain cancer, Electric fields, H3 K27 mutant

## Abstract

**Introduction:**

Diffuse intrinsic pontine glioma (DIPG) is a high fatality pediatric brain cancer without effective treatment. The field of electrotherapeutics offers new potential for other forms of glioma but the efficacy of this strategy has not been reported for DIPG. This pilot study evaluated the susceptibility of patient-derived DIPG cells to low intensity electric fields delivered using a developing technology called intratumoral modulation therapy (IMT).

**Methods:**

DIPG cells from autopsy specimens were treated with a custom-designed, in vitro IMT system. Computer-generated electric field simulation was performed to quantify IMT amplitude and distribution using continuous, low intensity, intermediate frequency stimulation parameters. Treatment groups included sham, IMT, temozolomide (TMZ) chemotherapy and radiation therapy (RT). The impact of single and multi-modality therapy was compared using spectrophotometric and flow cytometry viability analyses.

**Results:**

DIPG cells exhibited robust, consistent susceptibility to IMT fields that significantly reduced cell viability compared to untreated control levels. The ratio of viable:non-viable DIPG cells transformed from ~ 6:1 in sham-treated to ~ 1.5:1 in IMT-treated conditions. The impact of IMT was similar to that of dual modality TMZ–RT therapy and the addition of IMT to this treatment combination dramatically reduced DIPG cell viability to ~ 20% of control values.

**Conclusions:**

This proof-of-concept study provides a novel demonstration of marked DIPG cell susceptibility to low intensity electric fields delivered using IMT. The potent impact as a monotherapy and when integrated into multi-modality treatment platforms justifies further investigations into the potential of IMT as a critically needed biomedical innovation for DIPG.

## Introduction

Diffuse intrinsic pontine glioma (DIPG), also called H3 K27M-mutant diffuse midline glioma, is the most common brainstem cancer in children, representing 80% of tumors in this region of the central nervous system (CNS) [1, 2]. The average age at diagnosis is 6–9 years old and affected children typically present with a progressive spectrum of neurological deficits. Magnetic resonance imaging most often reveals an expansile, T2-hyperintense, poorly-enhancing mass centered in the pons [3]. The infiltration of DIPG within the brainstem parenchyma precludes safe surgical resection. The mainstay of treatment is fractionated radiation therapy (RT) which can provide transient symptom and tumor control resulting in median patient survival of 9 months and a 2-year survival rate < 10% [4]. Multiple chemotherapy protocols and agents have been trialed but have not demonstrated further survival benefit [5, 6]. Despite international efforts in defining the molecular underpinnings of DIPG, there have been no recent therapeutic advances that substantially improve patient outcomes [2, 4].

There is emerging evidence that electrotherapy may offer a novel means to control malignant glioma and considerable progress has been made treating the high fatality CNS cancer, glioblastoma (GBM). A single clinical electrotherapy system with demonstrated survival benefit has been approved for new and recurrent GBM. Unfortunately this device is not engineered for the treatment of infratentorial tumors such as DIPG [7–9]. Current PubMed searches using the terms diffuse intrinsic pontine glioma and electrotherapy or electric fields produced no reports. Thus, the potential impact of electrotherapy on DIPG remains unknown. Intratumoral modulation therapy (IMT) is a developing technology which may offer an electrotherapeutic option for tumors located anywhere within the CNS, including the brainstem [10, 11]. IMT exploits the electrosensitivity of cancer cells using implanted field-generating sources (e.g., bioelectrodes) to deliver non-ablative, low intensity electric fields that attenuate tumor growth and bolster multi-modality treatment platforms. Preclinical studies demonstrated potent efficacy of IMT as a monotherapy and when combined with chemotherapeutic or oncogene-silencing agents in primary human GBM cells and in vivo allogeneic GBM models [10, 11]. To date, however, there have been no reports of the application of IMT to other CNS cancers. The goal of this pilot study was to determine the vulnerability of patient-derived DIPG cells to low intensity electric fields delivered using an established IMT protocol. The impact of IMT on DIPG resistance to conventional radiation and chemotherapy options was also investigated.

## Materials and methods

### Patient-derived DIPG cells

Patient-derived DIPG cells, labeled SU-DIPG-IV, SU-DIPG-XIX and SU-DIPG-XXIV, were kindly received from Dr. Michelle Monje at Stanford University. The tumor collection protocol, culture methods at derivation, patient demographics and tumor genetics have been previously described [12–14]. The cells were derived from early postmortem DIPG specimens in three pediatric patients, aged 2, 2 and 6 years old respectively, who had received radiation and chemotherapy during their care. Upon arrival to the Hebb lab, frozen DIPG cells were thawed and cultured in 6-well plates (Corning, NY, United States) at 37 °C with humidified air containing 5% CO_2_ using NeuroCult™ NS-A medium supplemented with 10% proliferation supplement, bFGF (10 ng/mL), EGF (20 ng/mL), heparin sulfate (2µg/ml) and 1% penicillin/streptomycin (*Complete NSA*; Stemcell Technologies, Vancouver, B.C., Canada). The medium was changed every 72 h and cultures passaged 1:2 at 70–90% confluence.

### In vitro IMT model

The impact of IMT alone and combined with temozolomide (TMZ) chemotherapy and/or RT was evaluated using primary human DIPG cells cultured in 35 mm wells. The IMT model was created by fitting each well with a clinical grade, platinum-based strip bioelectrode (Ad-Tech Medical Instrument Corporation, Oak Creek, WI, USA) around the periphery and platinum-iridium bioelectrode (Medtronic Ltd., Hertfordshire, United Kingdom) at the center of the well and cell culture [10, 11]. A waveform generator (Rigol DG1022; Electro-Meters Ltd., Pickering, ON, Canada) was used to deliver biphasic sinusoidal pulses with low amplitude (± 2 V; peak-to-peak 4 V) and intermediate frequency (200 kHz) continuously over 3 days. Control wells (i.e. sham-treated) were fitted with bioelectrodes but did not receive stimulation.

### IMT field simulation

The IMT electric field was simulated with COMSOL Multiphysics v 5.2a (Comsol Inc., Burlington, MA, USA) AC/DC module, electric currents user interface in the frequency domain. The geometries of the in vitro model were manually created utilizing COMSOL Geometry to generate an identical in silico model. The dish and electrode apparatus were mimicked in geometry and the appropriate materials simulated utilizing known material conductance and dielectric properties. The boundary conditions were applied as the edge of the 35 mm dish. The centre stimulating electrode was set to emit a 2 V amplitude waveform, and the outer electrodes were grounds. Electrical insulation on outer boundaries was assumed. A mesh of the geometry was created, and a frequency of 200 kHz chosen for evaluation. The dimensions and amplitude of the electric field within the culture dish were analyzed using MATLAB R2015b (MathWorks Inc., Natick, MA, United States).

### Multi-modality treatment of DIPG cells

DIPG cells (1 × 10^5^) treated with 50 µm TMZ were cultured in 35 mm wells fitted with IMT bioelectrodes without stimulation (i.e., sham IMT) for TMZ monotherapy or while receiving concurrent IMT during dual therapy. This TMZ concentration corresponds to plasma levels obtained with 150 mg/m^2^ in the adjuvant treatment phase of other forms of glioma [15]. Additional DIPG samples received 4 Gy of ionizing radiation in a single fraction using a Cobalt-60 irradiator with average dose rate of 74 cGy/min. This dose was chosen after a RT dose–response study to permit evaluation of cooperative benefits between different therapeutic modalities in vitro [16, 17]. The cells were allowed to recover for 1 h after RT and were then plated into 35 mm wells fitted with the IMT system. At this point, DIPG cells were cultured with no further treatment (i.e., RT monotherapy group), or in the presence of TMZ (50 µM) or IMT (i.e., TMZ-RT or IMT-RT dual therapy groups), or both TMZ and IMT (i.e., TMZ-RT-IMT multi-modality therapy group) for 3 days.

### Cell viability assay

Cell viability was evaluated using the 3-(4,5-dimethylthiazol-2-yl)-2,5-diphenyltetrazolium (MTT) spectral analysis (Sigma Aldrich, St. Louis, MO, United States). This colorimetric assay measures the reduction of yellow MTT by mitochondrial succinate dehydrogenase to an insoluble, dark purple formazan product. Immediately following the DIPG cell treatments described above, MTT (80 µl at 5 mg/ml) was added to the 35 mm wells and incubated for 3 h at 37 °C in a humidified 5% CO_2_ atmosphere. The cells were then lysed to release the purple formazan product by the addition of 300 µl dimethyl sulfoxide for 15 min at room temperature. Absorbance was measured using an Epoch microplate spectrophotometer (BioTek, Winooski, VT, United States). Cell viability was estimated using optical density values at 570 nm with references at 655 nm detected in each well. Brightfield images of cells stained with MTT were obtained using a Motic AE31 inverted microscope fitted with an Infinity 1–3 scientific complementary metal-oxide semiconductor camera (Lumenera Corp, Nepean, ON, Canada).

### Flow cytometry

Annexin V apoptosis detection with zombie red (ZR) was used to quantify fractions of live, apoptotic and dead DIPG cells, as per the manufacturer’s instructions (BioLegend, San Diego, CA, USA). Cell fractions were analyzed using a Becton Dickinson LSR II SORP flow cytometer running FACSDiva software (BD Biosciences, Mississauga, ON, Canada). Cells were first gated on forward scatter (FSC-) versus side scatter (SSC-) characteristics before excluding doublets using consecutive gating FSC-Area versus FSC-Width and SSC-Area versus SSC-Width plots. The populations of annexin V+/ZR−, annexin V+/ZR+, annexin V−/ZR+ and annexin V−/ZR− were then calculated with quadrant gates. Approximately 25,000 single cells were acquired per sample at a maximum event rate of 5000 events per second. Data were analyzed using FlowJo v 9.6.3 software (TreeStar Inc., Ashland, OR, USA).

### Statistical analysis

A *t* test was used to compare paired data sets. Multiple pairwise comparisons were performed using one-way analysis of variance (ANOVA) followed by Tukey post hoc analysis (SPSS Inc., Chicago, IL, USA). Data are presented as mean ± standard deviation with significance assumed at p < 0.05.

## Results

### IMT field mapping for in vitro DIPG cell treatment

**Fig. 1:**
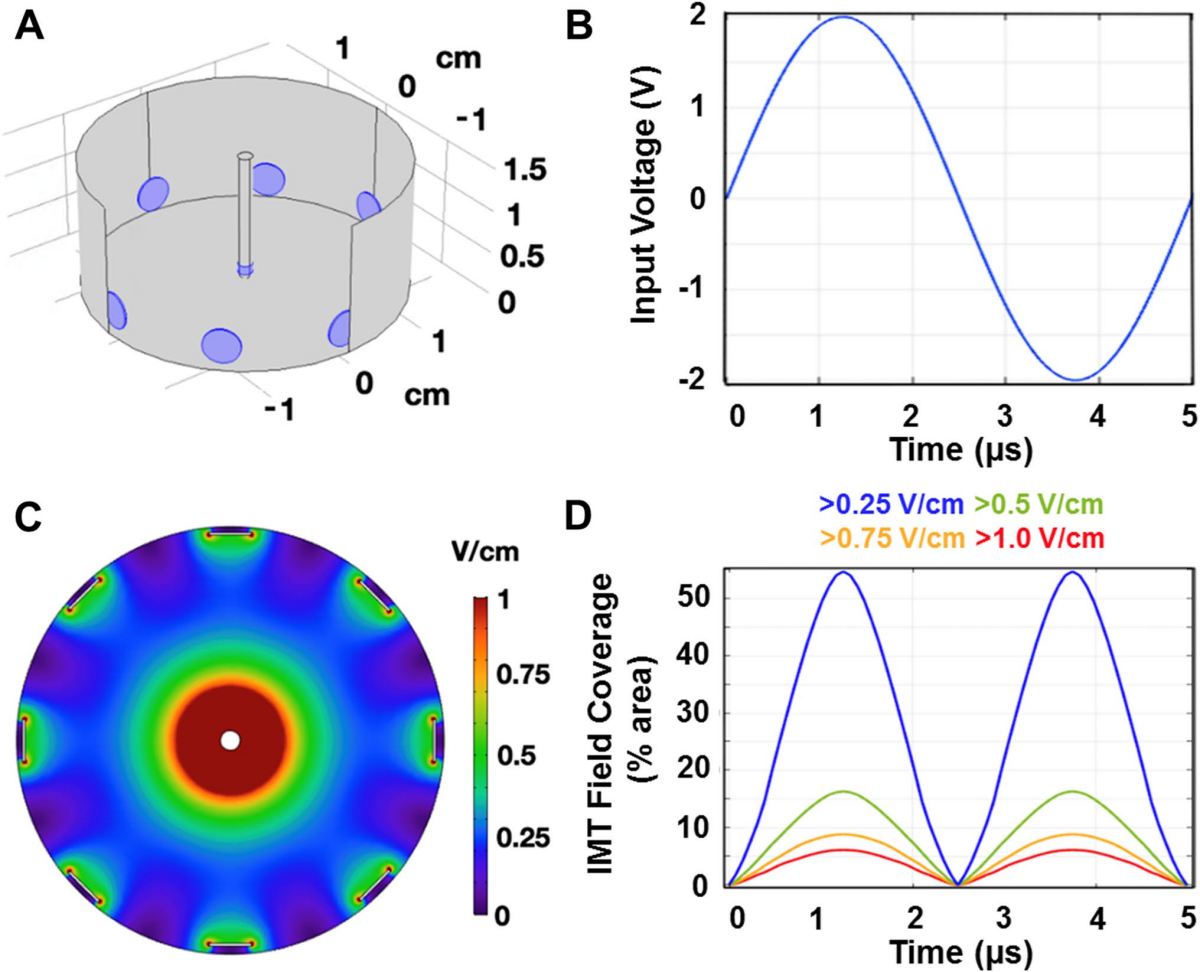
Computer simulation of the IMT model. **a** The IMT model was created using a central stimulating bioelectrode placed in a 3.5 cm well along with eight peripheral grounded bioelectrodes. **b** A waveform generator supplied a 200 kHz sinusoidal voltage with constant amplitude of 2 V to the central electrode. **c** Applying such parameters to this configuration of bioelectrodes resulted in an alternating electric field with the predicted magnitude and distribution plotted as shown. **d** The percent area coverage across the culture dish reaching the electric field thresholds in the range anticipated to exert biological impact are plotted as a function of time over one cycle of the waveform. This pilot IMT system provided only fractional field coverage to the DIPG cultures

The in vitro IMT model utilized clinical grade, biocompatible, platinum (peripheral ground) and platinum-iridium (central stimulating) bioelectrodes fitted within 35 mm culture preparations of patient DIPG cells (Fig. 1a). A sinusoidal, biphasic waveform with peak-to-peak amplitude of 4 V was chosen to create reversing polarity and maximally disrupt the electrical environment using low intensity parameters known to be innocuous within the living brain (Fig. 1b) [11]. The 200 kHz stimulation frequency is below that needed to produce thermal injury and surpassed the neuronal entrainment threshold to reduce the potential of off-target IMT effects when translated to eloquent brain regions [18–20]. The bioelectrode configuration and stimulation parameters created a symmetric, low intensity IMT field pattern across the DIPG cultures. The electric field was calculated by simulating the in vitro experiments in COMSOL Multiphysics (v 5.3a) using the electrode geometry presented and a constant voltage amplitude waveform generation. Based on this simulation, the largest electric field coverage extended concentrically from the central stimulating bioelectrode with smaller fields generated at regular intervals around the encircling peripheral bioelectrodes (Fig. 1c). The percent area coverage across the culture dish was calculated and plotted over one cycle of the waveform for electric field magnitude with thresholds in the range previously shown to be effective in other glioma cancers (Fig. 1d) [10, 11, 21, 22]. The coverage at the peak of the IMT waveform for each of the electric field magnitude thresholds of 1, 0.75, 0.5 and 0.25 V/cm was 6.2%, 8.9%, 16.4% and 54.7% respectively. Although other forms of glioma have been shown to require electric field amplitude > 1 V/cm for optimal electrotherapy benefits, the threshold for DIPG response is not known [21, 22]. The therapeutic effects described below were therefore generated with this pilot IMT model providing electric field coverage > 0.25 V/cm to roughly half, and 1 V/cm to only a small fraction of the DIPG culture area.

### Patient DIPG cells are vulnerable to low intensity IMT fields

**Fig. 2:**
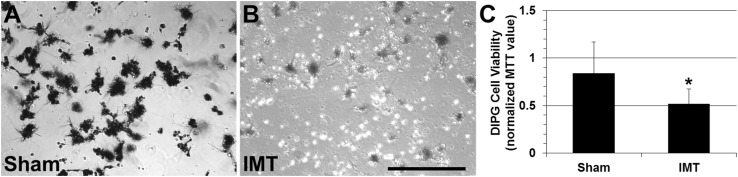
DIPG cells are highly susceptible to low intensity IMT. Representative brightfield microscopy showing patient DIPG cultures following a 3-day exposure to **a** sham or **b** IMT conditions. The cultures were stained with the viability dye, MTT which produces a dark chromogen in viable cells. IMT-treated DIPG cells exhibited marked pyknosis with reduced MTT labeling and density. **c** MTT measures in sham and IMT treated cultures were normalized to those obtained in parallel cultures of untreated DIPG cells. The IMT exposure produced a marked and significant reduction in DIPG cells (asterisk; p = 0.046; n = 3; mean ± standard deviation). Scale bar represents 500 µm for **a** and **b**

**Fig. 3:**
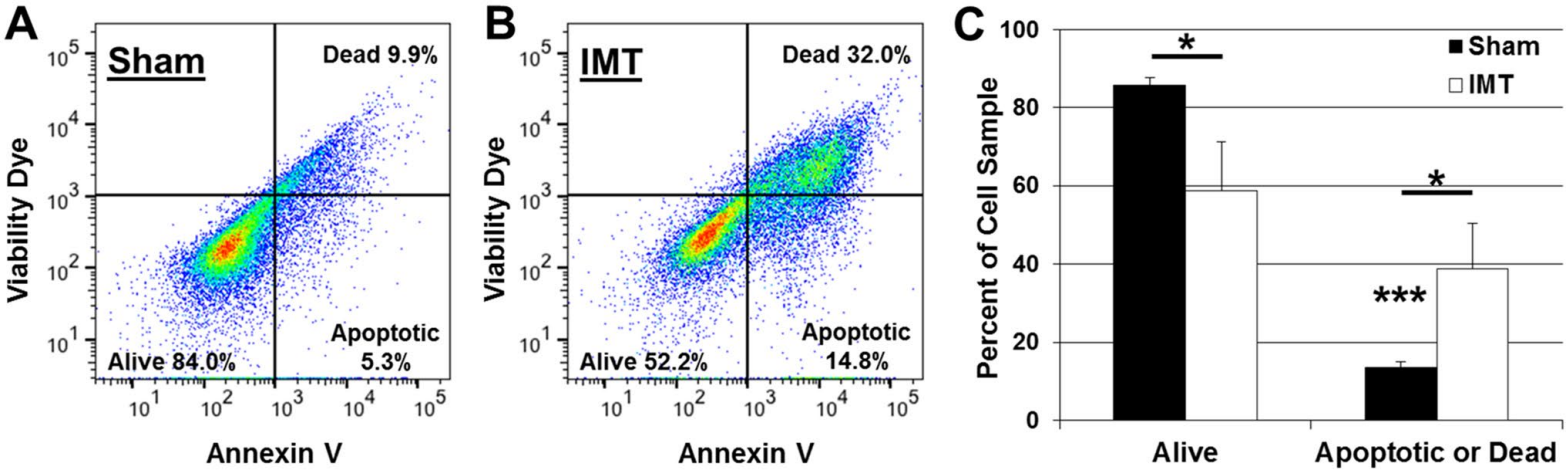
IMT enhances apoptosis and death fractions in DIPG cell cultures. **a** Representative flow cytometry scatterplots of annexin and the viability dye zombie red (ZR) labeling of apoptotic and dead DIPG cells, respectively, after a 3-day exposure to **a** sham or **b** IMT conditions. **c** Quantification of live and combined apoptotic/dead DIPG cell fractions. In the sham-treated cultures, there was a marked, significant discrepancy between these fractions that was attenuated in IMT-treated cultures owing to the significant rise in cell death. Asterisks immediately above the histogram bars indicate significance between the live and apoptotic/dead fractions within the same treatment group. Significance between indicated data pairs is depicted by the asterisk above the horizontal bars. Data are presented as mean ± standard deviation with significance indicated at P: * < 0.05 and *** < 0.001

Paired sham and IMT conditions were used to independently assess the impact of IMT monotherapy on primary DIPG cells (SU-DIPG-IV, SU-DIPG-XIX, SU-DIPG-XXIV) obtained from three pediatric patients. The 3-day exposure to low intensity IMT produced a dramatic and consistent reduction in tumor cell viability. IMT-treated cultures were sparse, pyknotic and exhibited faint MTT (formazan) labeling compared to sham-treated DIPG cultures (Fig. 2a, b). Statistical assessment performed on MTT measures normalized to those obtained in DIPG cells not exposed to IMT hardware (i.e., untreated controls) revealed a significant reduction in viability following IMT (51.6 ± 16.0%) compared to sham (84.0 ± 33.0%) treatment (p = 0.046; Fig. 2c). Flow cytometry was performed to confirm the MTT findings and evaluate the potential of apoptosis as a mechanism of IMT effect on DIPG cells. The ratio of viable:non-viable DIPG cells decreased from ~ 6:1 in sham-treated to ~ 1.5:1 in IMT-treated conditions (Fig. 3). The significant reduction in DIPG cell viability was accompanied by a concordant rise in apoptotic and dead cell fractions across all patient samples (Table 1).

**Table 1:** Summary of flow cytometry data in patient DIPG cell samples

DIPG # and cell fraction		SU-DIPG-IV			SU-DIPG-XIX			SU-DIPG-XXIV		
		Live (%)	Apoptotic (%)	Dead (%)	Live (%)	Apoptotic (%)	Dead (%)	Live (%)	Apoptotic (%)	Dead (%)
Treatment group	Sham	84	5.3	9.9	87.6	10	2.34	85.7	12.6	0.6
	IMT	52.2	14.8	32	73	18.7	6.9	51.2	32.2	11.7

### IMT significantly enhances multi-modality treatment platforms for DIPG

**Fig. 4:**
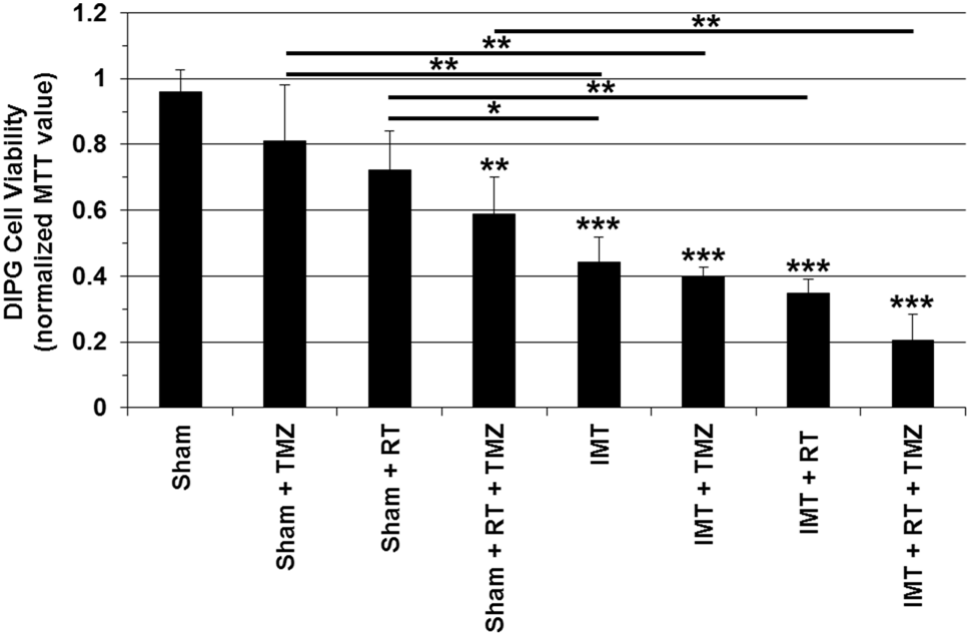
IMT significantly augments multi-modality treatment platforms for DIPG. TMZ, low dose RT and IMT were applied to patient DIPG cultures in 3-day mono- and multi-modality therapies. The MTT viability assay was used to evaluate the impact of each treatment and the resultant data normalized to the mean MTT value obtained in untreated control DIPG cells. Monotherapy TMZ and RT were delivered in culture wells fitted with sham IMT hardware and neither had a significant impact on DIPG cells. In contrast, dual TMZ-RT exposure dramatically reduced DIPG cell viability. The IMT effect did not differ significantly from that of dual TMZ-RT therapy. However, the addition of IMT to the TMZ-RT combination produced marked and highly significant added therapeutic impact and a resultant DIPG viability of approximately 20% of untreated controls. Asterisks immediately above the histogram bars indicate significance between that specific treatment group and sham control. Significance between indicated data pairs is depicted by the asterisk(s) above the horizontal bars. Data are presented as mean ± standard deviation with significance indicated at P: * < 0.05, ** < 0.01 and *** < 0.001

The impact of IMT monotherapy was compared to single agent TMZ chemotherapy and RT, as well as combined approaches using dual or triple modality platforms (Fig. 4). DIPG cells were treated with TMZ at a concentration equivalent to that achieved in plasma during standard care for GBM, another form of high grade glioma, with known modest, yet significant therapeutic efficacy and an acceptable toxicity profile [15]. The applied RT dose (4 Gy) in this study is known to have minimal impact in DIPG cells when used as a monotherapy, based on in-house experience (unpublished) and previous reports by other groups [16, 17]. This dose was chosen to determine if the negligible efficacy could be bolstered by the addition of TMZ and/or IMT to lessen the potential for adverse radiation effects when delivered in vivo. MTT analysis was used to assess the viability of DIPG cells following parallel 3-day exposures to sham IMT (i.e., hardware placement without stimulation) or IMT with or without TMZ, RT or combined TMZ-RT. All MTT measures were normalized to those of untreated DIPG controls. The sham IMT conditions had little effect on DIPG cells and yielded 96.0 ± 6.6% viability. Monotherapy TMZ and RT was assessed in culture wells fitted with a sham IMT system. TMZ (81.1 ± 17% viability; p = 0.564) and RT (72.2 ± 11.9% viability; p = 0.105) produced slight reduction in MTT values that were not statistically different from the sham-only controls. In contrast, the combined TMZ-RT exposure (which also included the sham IMT hardware) produced a mean DIPG cell viability of 58.9 ± 11.3% which was significantly reduced relative to the sham-only treated cells (p = 0.004). IMT monotherapy resulted in DIPG cell viability of 44.2 ± 7.7% which was also significantly reduced compared to sham values (p < 0.001) and significantly more effective than either TMZ (p = 0.005) or RT (p = 0.045) conditions. Dual therapy created by the addition of IMT significantly reduced DIPG cell viability compared to monotherapy TMZ (81.1 ± 17% vs. 40.1 ± 2.50%; p = 0.002) or RT (72.2 ± 11.9% vs. 34.7 ± 4.4; p = 0.004). In contrast to TMZ and RT monotherapies, the MTT-measured impact of IMT alone was not significantly different than the combined TMZ-RT treatment (p = 0.614). Likewise, the therapeutic effect of combined TMZ-RT exposure was not significantly different from dual modality exposure to IMT-TMZ (p = 0.315) or IMT-RT (p = 0.113). Most notably, however, the impact of dual therapy TMZ-RT was potently enhanced by the addition of IMT in the multi-modality treatment platform TMZ-RT-IMT (58.9 ± 11.3% vs. 20.50 ± 7.8% viability, respectively; p = 0.004).

## Discussion

This study provided novel evidence of DIPG cell vulnerability to low intensity electric fields delivered using an established preclinical IMT protocol. IMT produced apoptotic tumor cell death using stimulation parameters previously demonstrated to be non-injurious to normal neural cells in vitro and in vivo [10, 11]. The applied stimulation frequency of 200 kHz is above the range for neural entrainment and below that for thermal injury [19, 20]. IMT monotherapy reduced DIPG cell viability by ~ 50% and was more efficacious than either low dose RT or a clinically utilized concentration of TMZ. The addition of IMT to the combined TMZ-RT paradigm dramatically reduced DIPG cell viability from ~ 60% to 20%. It remains unclear how IMT incites glioma cell death. There is evidence that non-ablative, non-thermal, low intensity electric fields work through multiple anti-neoplastic mechanisms that disrupt polarized molecules necessary for cell division, membrane permeability and channel homeostasis [23, 24]. In GBM studies, IMT has been shown to enhance the impact of TMZ and oncogene-targeted therapy as well as the uptake of hydrophilic genetic inhibitors [10]. The apparent lack of effect on post-mitotic neurons or in normal brain parenchyma suggests that IMT exploits electrochemical vulnerabilities related to the neoplastic phenotype. Further studies are required to better understand these issues.

One possible method of providing IMT to DIPG entails custom configured, implanted bioelectrodes to perpetually deliver therapeutic electric fields across tumor-affected CNS regions using a concealed, titratable system that works cooperatively within a multi-modality treatment platform. Radiation is currently first line care for DIPG and, in this study, produced cooperative anti-neoplastic impact when delivered concurrently with IMT [4]. While chemotherapy has not shown significant benefit in DIPG for a potential host of reasons, TMZ is commonly used and beneficial in other forms of malignant glioma [6, 15]. In this study, the insignificant impact of RT or TMZ monotherapies was in contradistinction to the potent effect of the triple IMT/RT/TMZ combination, suggestive of sensitizing interactions that dramatically improve overall treatment efficacy. It remains to be determined if this efficacy will persist in vivo, and whether the multi-modality platform brings any unexpected toxicities. With respect to safety, a key putative advantage of the envisioned clinical IMT system is the ability to titrate, re-configure and discontinue stimulation. Reversibility of effect with therapy cessation, as typical with conventional forms of neuromodulation such as deep brain stimulation (DBS), is likely given the low amplitude and apparently benign nature of applied IMT fields in normal brain [11, 30–32].

The most readily available IMT prototype will likely include multi-contact leads stereotactically positioned within the brainstem and powered by an indwelling pulse generator. Surgical access to the brainstem is routinely achieved for various neoplastic and non-neoplastic indications and does not present an obvious barrier to development and safe application of IMT for DIPG patients. For example, there has been recent progress directly targeting DIPG tumors with catheters used for convection-enhanced delivery (CED) of pharmacotherapies [25, 26]. While the outcome of the neoplastic disease was not altered using CED, there were no significant adversities that resulted from implanting catheters within the DIPG tumors. A longstanding, global experience with DBS provides another important example of the feasibility to implant hardware in the brainstem for treating neurological disease. DBS is the standard of surgical care for Parkinson’s Disease (PD) where electrodes are chronically implanted into the subthalamic nucleus and often extending into the mesencephalic substantia nigra [27, 28]. The pedunculopontine nucleus is another brainstem target being evaluated for implanted neuromodulation systems to improve postural and gait instability in PD [29]. It is important to note that DBS and IMT are starkly different in neurological indication, operational parameters and hardware design. The electrical output of putative IMT systems will be defined by customized waveform, polarity, and stimulation parameters titrated to individual tumor response and regional anatomy. DBS technology typically delivers continuous, monophasic, square wave pulses at low frequency (e.g., 90–185 Hz) to disrupt and entrain pathological firing patterns [30–32]. In contrast, the present IMT system used an intermediate frequency (200 kHz), sinusoidal waveform with reversing polarity intended to maximally disrupt electrical homeostasis in DIPG cells. The pulse frequency was 1000-fold higher than typical DBS settings and well out of range for neuronal entrainment in order to selectively target neoplastic cells while averting adverse neurological effects when stimulating tumor-infiltrated CNS regions [11, 18, 33]. Additionally, the hardware configuration required to deliver personalized, comprehensive IMT field coverage will likely be sharply divergent from that of contemporary DBS electrodes designed to provide highly discrete zones of stimulation.

This proof-of-concept study provided novel evidence of DIPG cell susceptibility to low intensity electric fields delivered using an IMT strategy. The significant treatment response was produced in a pilot IMT model with incomplete field coverage, suggesting even greater efficacy may be realized using a comprehensive delivery system. Additionally, these DIPG cells were derived from fresh autopsy tissue that had been previously exposed to chemotherapy and RT, possibly selecting for tumor cells with heightened treatment resistance. The impact of IMT-based platforms measured in this study may therefore underestimate that attainable in treatment-naïve DIPG cells. While the present observations have yet to be replicated in larger, genetically-diverse cohorts and translational models, these exciting early data provide a new glimpse at the potential of electrotherapeutics to improve the otherwise devastating outcome for DIPG patients.
